# Survey on the Prevalence of Allergic Rhinitis and its Effect on the Quality of High School Students’ Life 

**Published:** 2013

**Authors:** Maryam Amizadeh, Hossein Safizadeh, Nasrin Bazargan, Zahra Farrokhdoost

**Affiliations:** 1 *Department of Otorhinolaryngology, Kerman University of Medical Sciences, Kerman, Iran.*; 2 *Neurosciences Research Center, Kerman University of Medical Sciences, Kerman, Iran.*; 3 *Department of Pediatric, Kerman University of Medical Sciences, Kerman, Iran.*; 4 *Shafa Hospital, Kerman University of Medical Sciences, Kerman, Iran.*

**Keywords:** Allergic rhinitis, Kerman, Quality of Life, SF-36, SFAR

## Abstract

**Introduction:**

Allergic rhinitis (AR) is a common airway disease. In order to study the prevalence of AR in high school students in Kerman, the Score for Allergic Rhinitis (SFAR) was used and the quality of life in the students affected by rhinitis was evaluated using the SF-36 questionnaire.

**Materials and Methods:**

This was a cross-sectional, analytical, descriptive study, based on the SFAR scale. Quality of life in students with AR was evaluated using the SF-36 questionnaire.

**Results:**

From 1511 students who completed the SFAR questionnaire, 291 (52.6%, girls; 47.4%, boys) had AR. Domestic dust was the most common cause of the disease. The most common symptoms of AR were rhinorrhea (76.6%), epiphora (76.3%), nasal congestion (64.3%), and itching (54.3%). According to the ARYA scale, (Allergic Rhinitis and its Impact on Asthma), 41.9% of students had moderate-to-severe rhinitis and 58.1% had mild rhinitis. A total of 43.1% of patients with moderate-to-severe rhinitis had a persistent condition and 56.9% had an intermediate condition. Results of the SF-36 questionnaire among students with AR showed a significant difference in physical functioning and bodily pain in comparison with healthy students.

**Conclusion:**

The results of this study show that the prevalence of AR among Kerman high school students is 19.3%. Because of the effect of this disease on the life quality of high school students in terms of both physical functioning and bodily pain, efforts should be made to reduce allergen levels as far as possible.

## Introduction

Allergic rhinitis (AR) is an upper respiratory disease caused by immunoglobulin E (IgE) interference following contact with allergens (1). Allergic rhinitis may be classified as seasonal, perennial, or episodic. In seasonal AR, symptoms occur during exposure to seasonal allergens such as pollen, while in the case of perennial AR, symptoms may last for 2 hours a day and more than 9 months a year. Symptoms in episodic rhinitis occur due to exposure to allergens which are not normally present, including allergens from cats and other pets (2).

Symptoms of AR usually appear before the age of 20 (2), and may limit the sufferer’s daily activities. Each year specialists see a high burden of symptoms of AR (including cough, itching, rhinorrhea, epiphora, and congestion) among patients, resulting in high treatment costs (1). 

To date, a number of studies have been undertaken in Iran investigating the prevalence of AR. One such study performed in Hamadan reported an AR prevalence among high school students of 34.3% (3), while a similar prevalence of 35.3% among 12–13 year-olds was reported in the city of Gorgon (4). An AR prevalence of 39 among 4959 individuals (3%) was reported in Belgium (5), while of 1200 individuals over the age of 14 in the UK, 16 (8%) and 19 (8%) had abstractive and itching symptoms, respectively. 

As the Kerman geographical zone is surrounded by a dry climate as well as agricultural land, and because a large number of allergens have been recorded in the area, we decided to investigate the prevalence of AR in this city.

## Materials and Methods

This was a descriptive, analytical, cross-sectional study. The population of this study was high school students in the city of Kerman.

The survey was carried out in two phases. In the first phase, the prevalence of AR was investigated; while in the second phase, the quality of life of students with AR was compared with that of healthy students.

In the first phase, 1840 students selected through cluster sampling were investigated. Sixteen schools were chosen at random among Kerman high schools, and all students from one class at each level of study were chosen at random and questioned. All students in the study were given the project questionnaire. 

The questionnaire consisted of three parts. In the first part, the demographic characteristics of the students were registered, while the second part included the score for AR (SFAR) scale. This scale runs from 0 to 16, with a sensitivity of 74% and a specificity of 83% for SFAR≥7, satisfactorily discrimi- nating between outpatients with AR and those without AR. The advantage of this scale is that it requires only a short time for completion, and is easy to understand (7). In the continuation of the questionnaire, questions were asked concerning contact with tobacco smoke and opium. In the third and final part of the questionnaire, the ARYA scale was used to determine the severity of rhinitis. In this scale, students were divided into four groups; mild persistent, moderate-severe persistent, mild intermittent, or moderate-severe intermittent AR. An intermittent rating indicated that symptoms were observed on fewer than 4 days a week or for less than 4 weeks. In addition, patients were divided into mild and moderate-to-severe groups according to patterns of sleep and daily activities (12).

In the second phase of the research, and in order to study the quality of life of students with AR, the SF-36 questionnaire was used. This questionnaire investigates quality of life in eight domains, including physical functioning, role limitation due to physical problems, general health, vitality, social functioning, mental health, role limitation due to mental problems, and bodily pain. The quality of life score in each domain ranges between 0 and 100. A score close to zero indicates a poorer quality of life, while a score close to 100 indicates better quality of life. The Farsi equivalent of this questionnaire was available and its validity and reliability were approved (8). Students with AR were selected for the questionnaire using simple sampling by referring to the available samples, while healthy classmates were used as controls. 

For statistical analysis, t-test and Chi-Square tests were performed, using SPSS 11.5 software. 

## Results

In the first phase of this study, 1840 questionnaires were distributed, of which 1511 (82%) were completed and returned. Respondents included 825 (54.6%) girls and 686 (45.4%) boys, all of whom were high school students. 

Based on the SFAR scale, 291 students (19.3%) had AR, of whom 153 (52.6%) were girls and 138 (47.4%) were boys. Symptoms during the preceding 12 months among students with AR included nose itching (54.3%), congestion (64.3%), and rhinorrhea (76.6%). The rate of eye itching and epiphora was 76.3%. There was a significant difference in symptoms compared with healthy students (P< 0.001). Of the students with AR, 176 (61.6%) were aware of their allergy, with 38 (13.1%) already having had an allergic test and 33 (87%) having received positive results. Asthma had already been diagnosed in 18.6% of cases, compared with eczema in 4.1% of cases and AR in 12% (P<0.001 versus healthy controls). Compared with healthy families, these diseases were more common among individuals with a family history of allergy (P<0.001). However, students with an allergy background had greater contact with tobacco smoke and opium compared with healthy controls (P<0.001). A comparison of AR symptoms between students with and without AR is shown (Table 1).

**Table 1:** Comparison of Allergic Rhinitis (AR) symptoms between students with AR and without AR

	**Without AR** **N (%)**	**With AR** **N (%)**	**P value**
Symptom			
Runny nose	115 (9.4)	158 (54.3)	<0.001
Blocked nose	187 (15.3)	187 (64.3)	<0.001
Sneezing	324 (26.6)	223 (76.6)	<0.001
Itchy watery eyes	212 (17.4)	222 (76.3)	<0.001
Previous history			
Asthma	37 (3)	54 (18.6)	<0.001
Eczema	9 (0.7)	12 (4.1)	<0.001
AR	3 (0.2)	35 (12)	<0.001
	85 (7.3)	99 (35.6)	<0.001
Contact with smoking	262 (22.6)	93 (33)	<0.001

In students with AR, 122 (41.9%) had moderate-to-severe disease and 169 (58.1%) had mild disease. However, 43.1% of those with moderate-to-severe AR had a persistent condition and 56.9% had intermediate symptoms. It was also calculated that among patients with mild rhinitis, 28.6% had a persistent condition and 71.4% had intermediate symptoms, showing significant differences among those with AR (χ=5.41, df=1, P<0.05). 

In the second phase of the study, the quality of life of the 126 students with AR as well as 133 healthy students was investigated using the SF-36 questionnaire. After calculating the scores, quality of life was studied across the eight domains, and it was determined that the lowest scores were related to general health then social functioning, while the highest score was related to physical functioning. A comparison of quality of life between students with and without AR is shown in Table 2. The only significant differences between the two groups relates to physical functioning and bodily pain. This comparison shows that the quality of life of the students with AR was worse than that of the healthy students (P<0.05) (Table 2).

**Table 2:** Comparison of quality of life between students with and without AR

**SF-36 Domain**	**Without AR mean (±SD)**	**With AR** **mean (±SD)**	**P Value**
Physical function	85.26 (19.51)	78.33(22.23)	< 0.05
Role limitation-physical	69.41(25.74)	65.60(22.93)	>0.05
Bodily pain	76.63 (25.22)	70.12(23.59)	<0.05
General health	53.79 (17.33)	52.6(15.56)	>0.05
Vitality	63.20 (18.43)	60.27(18.45)	>0.05
Social functioning	61.80 (18.78)	57.70(19.60)	>0.05
Role limitation-emotional	64.44 (27.23)	62.30(22.40)	>0.05
Metal health	66.32(22.00)	64.60(22.23)	>0.05

## Discussion

The main objective of this research was to study the prevalence of allergic rhinitis among high school students (15–18 years of age) in the city of Kerman (south east of Iran) using the SFAR scale. Based on this study, the prevalence of AR is estimated at 19.3%. This compares with other studies carried out in Iran using the International Study of Asthma and Allergic in Childhood protocol (ISSAC), which have previously show a prevalence of 17.7% in Hamadan (west of Iran) (9), 25.5% in Booster (south of Iran) (10), and 29.6% in Kashan (center of Iran) (11). The ISSAC method considers seasonal AR predominantly, while other types of rhinitis, such as perennial disease, are not studied (6). Thus, the difference in prevalence compared with the statistics obtained in the city of Kerman might be explained by weather changes and changes in allergen levels, or by the kind of questionnaire used.

In this study, there were no significant differences between the two sexes, while there was a significant difference between the two sexes in the study undertaken in Rasht (12). In the study conducted in Hamadan (9), there was no significant difference between the two genders, while in a study carried out by Hatami (10) in Boushehr and KHaldif (13), the prevalence was also equal between the two sexes. In other studies by Kao (14) and Oliveri (15), the prevalence of AR was greater among boys than girls. Furthermore, in our current study, patients with AR had had greater contact with cigarette and opium smoking, consistent with previous studies (9).

The SAFARI study conducted in Hamadan (9) showed that 37.2% of the samples had persistent AR, while a different study conducted in France showed a rate of persistent AR of 49% (16). Furthermore, in a study carried out in France among those with SFAR ≥ 7, 10% had mild intermittent AR, 14% had moderate-to-severe intermittent AR, and 59% had severe-to-moderate persistent rhinitis (17). In the present study, 41.9% of patients had moderate-to-severe rhinitis, of whom 43.1% had persistent AR. 

It has previously been shown that the quality of life at school of students with AR is lower than that of peers without AR, and it has also been estimated that AR is responsible for the loss of 3.5 million working days and 2 million school days each year (18). One study evaluated the impact of AR and asthma on quality of life, and proved that patients with AR experience problems with social and daily activities and have a lower mental well-being than patients without AR. Furthermore, patients with AR had poorer general health and lower vitality than subjects without AR (19). 

In our study the lowest score was related to general health and the highest score was related to physical functioning, and the only significant difference between groups related to physical functioning and bodily pain. These findings were in contrast with some other studies reported. Hellgren studied quality of life in noninfectious rhinitis (NIR) and demonstrated that NIR patients scored lower in vitality, physical function, and social function than healthy persons (20). Bunny estimated a difference between AR patients and controls in all SF-36 domains except Social Functioning dimension (21). Our results are most consistent with those of Hellgren. The differences between our study and others might be due to the use of different cultures as well as different problems and patient symptoms. 

## Conclusion

As the scales used in this study were not the same as the scales used in similar studies in other cities, the prevalence of rhinitis in Kerman cannot be compared directly with previous reports from other cities. However, considering the value of the scale used in this study and the prevalence calculated in this study, as well as our knowledge about the effect of this disease, we can state with some confidence that AR reduces physical function and increases bodily pain among young people.

As observed in this study, AR is highly dependent on smoking and opium habits. Therefore, reducing contact with these factors might reduce the allergic attacks which have such a negative impact on individuals’ performance. This finding highlights the negative consequences and high price associated with drug usage. 
